# Neural correlates of perceptual texture change during active touch

**DOI:** 10.3389/fnins.2023.1197113

**Published:** 2023-06-02

**Authors:** Jessica Henderson, Tyler Mari, Andrew Hopkinson, Danielle Hewitt, Alice Newton-Fenner, Timo Giesbrecht, Alan Marshall, Andrej Stancak, Nicholas Fallon

**Affiliations:** ^1^School of Psychology, University of Liverpool, Liverpool, United Kingdom; ^2^Hopkinson Research, Wirral, United Kingdom; ^3^Institute of Risk and Uncertainty, University of Liverpool, Liverpool, United Kingdom; ^4^Unilever, Research and Development, Port Sunlight, United Kingdom; ^5^Department of Electrical Engineering and Electronics, University of Liverpool, Liverpool, United Kingdom

**Keywords:** electroencephalography, texture perception, active touch, time-frequency analysis, change detection

## Abstract

**Introduction:**

Texture changes occur frequently during real-world haptic explorations, but the neural processes that encode perceptual texture change remain relatively unknown. The present study examines cortical oscillatory changes during transitions between different surface textures during active touch.

**Methods:**

Participants explored two differing textures whilst oscillatory brain activity and finger position data were recorded using 129-channel electroencephalography and a purpose-built touch sensor. These data streams were fused to calculate epochs relative to the time when the moving finger crossed the textural boundary on a 3D-printed sample. Changes in oscillatory band power in alpha (8–12 Hz), beta (16–24 Hz) and theta (4–7 Hz) frequency bands were investigated.

**Results:**

Alpha-band power reduced over bilateral sensorimotor areas during the transition period relative to ongoing texture processing, indicating that alpha-band activity is modulated by perceptual texture change during complex ongoing tactile exploration. Further, reduced beta-band power was observed in central sensorimotor areas when participants transitioned from rough to smooth relative to transitioning from smooth to rough textures, supporting previous research that beta-band activity is mediated by high-frequency vibrotactile cues.

**Discussion:**

The present findings suggest that perceptual texture change is encoded in the brain in alpha-band oscillatory activity whilst completing continuous naturalistic movements across textures.

## 1. Introduction

The human brain processes ongoing sensory information by comparing incoming sensory information to previous stimulation, which enables humans to detect changes in their environment (Laufer et al., 2008). As humans, we explore our haptic environment through active touch and the glabrous skin on our hands and digits (Gibson, 1962; Wagner and Gibson, 2016). Active touch is integral in identifying and evaluating objects and surfaces by optimising contact pressure, speed, and velocity (Lederman and Klatzky, 2009). The present study aimed to investigate how the brain encodes continuous tactile information associated with changes in touch experience by assessing neural oscillations related to perceptual texture change.

Processing of stimulus change in the brain has previously been investigated using electroencephalography (EEG) and oddball tasks (Naatanen et al., 1978), wherein participants are exposed to one repetitive stimulus and then presented with a novel oddball stimulus. This type of paradigm results in an event-related potential (ERP) response called mismatch negativity (MMN); a correlate of auditory change perception that peaks around 100–300 ms following novel stimuli (Näätänen et al., 2005, 2007). Though primarily studied in the auditory domain, MMN effects have been reported in other sensory modalities such as vision (Stefanics et al., 2014) and somatosensation (sMMN; Restuccia et al., 2007; Butler et al., 2011, 2012; Hu et al., 2013; Chen et al., 2014). Oddball paradigms present stimuli for fixed intervals with a period of rest prior to stimulation, thus revealing information about the brain processing of individual events and features. A drawback of this approach is that this type of stimulation is not representative of real-world experiences, where ongoing sensory processing occurs, and change detection is an additive experience rather than an isolated feature.

In contrast to ERP analysis, investigating neural oscillations using time-frequency analysis provides a more accurate representation of ongoing sensory processing in the brain, by offering insights into neuronal synchrony and ongoing inter-neuronal communication (Mathalon and Sohal, 2015; Morales and Bowers, 2022). This approach enables the investigation of the summation of neural oscillations and ongoing changes in the brain in response to external stimuli (Cohen, 2014). Alpha-band and beta-band oscillations are attenuated during tactile stimulation or voluntary movement (Chatrian et al., 1958; Pfurtscheller, 1981; Salmelin and Hari, 1994) over the primary somatosensory and motor cortices, respectively (Brovelli et al., 2004). Attenuation of alpha- and beta-band oscillations are thought to reflect increased cortical activation, whereas the presence of synchronous oscillations is indicative of cortical areas at rest (Pfurtscheller, 1992, 1999).

Texture processing in the brain manifests bilaterally as attenuation of alpha-and beta-band rhythms relative to a rest period (Genna et al., 2018; Henderson et al., 2022). Whilst active exploration of texture has been investigated with EEG (Henderson et al., 2022), the neural mechanisms that underpin the processing of texture change during naturalistic explorations are relatively unknown. Processing of rough and smooth textures demonstrate altered cortical responses, with smooth textures typically eliciting increased brain activation relative to rougher surfaces during passive tactile stimulation (Moungou et al., 2016; Genna et al., 2018). Comparatively, our recent study investigating the neural correlates of texture perception with active touch found increased attenuation of beta-band oscillations for smooth textures and increased attenuation of alpha-band oscillations for rough textures (Henderson et al., 2022). Taken together, the literature indicates that cortical changes which occur during texture processing are modulated by textural properties, such as surface roughness. Therefore, change detection in the brain during ongoing processing is also likely to demonstrate altered neural oscillatory response as an indication of encoding textural change.

Change detection in the brain has been previously investigated using time-frequency analysis of MMN ERPs, which revealed increased theta-band power across auditory (Fuentemilla et al., 2008; Hsiao et al., 2009; Ko et al., 2012), visual (Stothart and Kazanina, 2013; Liang et al., 2017), and somatosensory modalities during unattended pressure stimulation to the finger pad (Zhang et al., 2019). Theta-band oscillations have also been linked with top-down memory processes, which led to increased power in frontal midline regions (Klimesch, 1999; Klimesch et al., 2008). Further, ERPs in the time-domain are suggested to manifest as theta-band changes in the time-frequency domain (Bernat et al., 2007; Harper et al., 2014). Therefore, theta-band power may reflect event-related changes in neural processing of texture change. However, it should be noted that findings from oddball tasks are not representative of complex environmental change, and therefore oscillatory brain activity may differ under active ongoing exploration.

The present study utilised touch sensor technology to investigate cortical oscillatory changes in alpha-, beta- and theta-bands during active touch of two adjacent textures. A touch sensor was used to quantify touch behaviour in real-time and compute time markers from when the index finger crossed the point of texture transition. Time markers were subsequently integrated with EEG data to consider event-related changes in oscillatory activity. We hypothesised that brain oscillations would show differences related to texture change; specifically, there will be reductions in alpha- and beta-band power over sensorimotor areas related to perception of texture change. Further, we hypothesise that theta-band power would increase as an oscillatory reflection of perceptual change mechanisms in frontal regions. A secondary analysis compared texture change from rough-to-smooth, relative to smooth-to-rough transitions. We hypothesised beta-band power would decrease over sensorimotor areas when transitioning from rough to smooth, whereas alpha-band power would decrease when transitioning from smooth to rough, demonstrating texture differences in frequency bands in line with our previous research (Henderson et al., 2022).

## 2. Materials and method

### 2.1. Participants

Thirty-five participants were recruited with no history of any neurological condition, or aversion or allergies to any textures. Visual inspection of the data for the presence of any movement or muscle artefacts was conducted. Five participants were excluded due to over 45% of trials being marked for rejection or when over 10% of channels were interpolated. The final sample included 30 participants (12 males, 2 left-handed), aged 28.43 ± 5.05 years (mean ± SD). Participants were reimbursed at a rate of £10 per hour for their time. The study was approved by the Research Ethics Committee of the University of Liverpool and all participants gave fully informed written consent at the start of the experiment in accordance with the Declaration of Helsinki.

### 2.2. Procedure

Participants were seated in a dimly lit Faraday cage with a 19-inch LCD monitor approximately 1 m in front of them. The tactile contrast task and practise trials were presented using PsychoPy (Peirce et al., 2019). Six-axis touch sensor and EEG data were recorded during the tactile contrast task. An elbow rest was used to stabilise and support the arm whilst maintaining position over the measuring plate of a six-axis touch sensor. The height and position of the support were adjusted for each participant.

#### 2.2.1. Stimuli

Texture stimuli were designed using algorithms adapted from Kanafi (2022) implemented in MATLAB (The MathWorks, Inc., United States), which produce isotropic textures representative of rough surfaces found in the real-world with well-defined power spectral distribution (PSD) given by (Eq. (1)):

Equation 1. Isotropic texture calculation.


(1) $$\phi \left(\mathrm{||}q\mathrm{||}\right)=\{\begin{array}{l} C,\\ C\left(\frac{\left|q\right|}{q_{r}}\right)\\ O, \end{array}\begin{array}{r} -2\left(1+H\right)\;\\ , \end{array}\begin{array}{cc} \; & if\;q_{0}\leq \left|q\right|\leq q_{r}.\\ \; & if\;q_{r}\leq \left|q\right|\leq q_{1}.\\ \; & otherwise. \end{array}$$

where *C* is a constant which determines roughness amplitude, $q_{0}$ and $q_{1}$ define the lower and upper limits of the wavenumbers *q,* and $q_{r}$ is the wavenumber above which the power spectral density is reduced with increasing wavenumber at a rate which is dictated by *H*, the Hurst roughness exponent. For the texture used in the study, $q_{0}=1200$ rad/m, $q_{1}=3600\;$ rad/m and $q_{r}=2400$ . The corresponding range of wavelengths in the texture is from 2.62–5.24 mm. The constant *C* was adjusted to achieve an RMS surface roughness of 0.15 mm.

**Figure 1:**
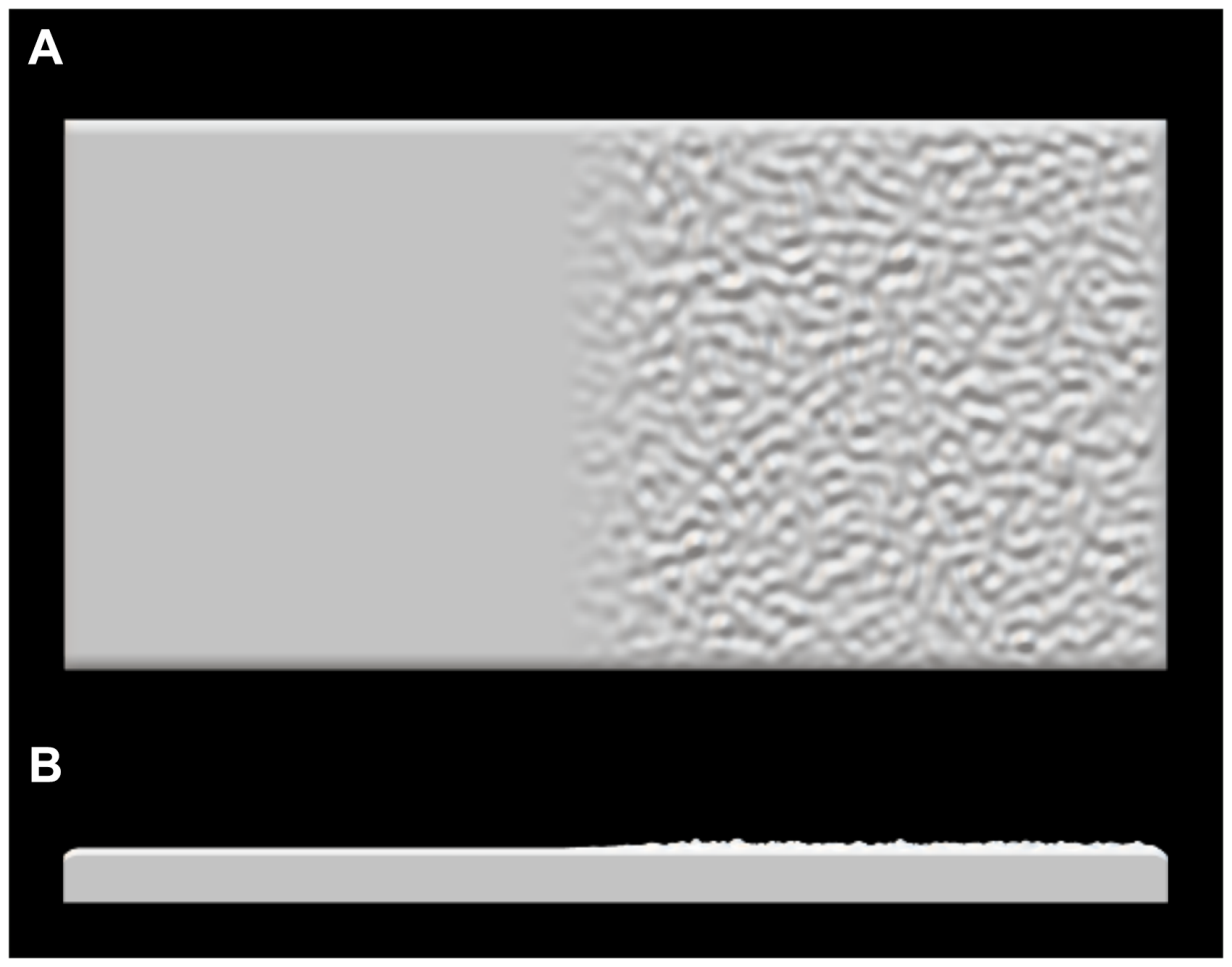
3D printed stimuli 100 mm × 50 mm where left is the smooth portion of the tile and right is the rough portion of the tile. Superior **(A)** and horizontal view **(B)**.

Textured stimuli were manufactured as a 100 × 50 mm resin tile, produced with a Formlabs Form 3 Stereolithography (SLA) 3D printer, where 40 mm of the tile was smooth and 40 mm was rough, with a 10 mm transition period in the centre where the two textures merged (Figure 1). During naturalistic texture exploration, humans typically use lateral movement across interior surfaces rather than edges (Lederman and Klatzky, 1987). Therefore, it was important the stimuli did not include a sharp edge between the textures, as the perception of edge properties is more indicative of structure-related perception, such as shape, rather than a textural change. Thus, the transition period was implemented in the MATLAB algorithm to reduce the sharp edge between the two stimuli to investigate the neural processing of texture rather than other haptic features. The tile was mounted on the measuring plate of the touch sensor in a landscape position.

#### 2.2.2. Tactile contrast task

The tactile contrast task comprised of four blocks, each lasting approximately 2 min. Participants were instructed to use a unilateral movement with the distal phalanx of their right index finger to complete sweeps across the texture. An 18 mm circle, the average width of a human index fingertip (Dandekar et al., 2003), was displayed on the screen to denote a finger sweep across a 10 cm line, in concordance with the 3D printed sample size. The dot moved across the plane at 2.5 cm/s; therefore, the index finger completed one sweep of the 3D texture in 4 s. Participants were trained to perform this movement prior to the experimental task by following the same visual cues whilst exploring an entirely smooth tile. Trials lasted 4 s and were performed back-to-back in a continuous block, meaning each block contained both smooth-to-rough transitions and *vice-versa*. Each block contained 30 trials (finger sweeps), totalling 120 trials over the experiment (60 trials per condition). Blocks were counterbalanced by starting texture (i.e., starting on smooth or rough). Participants kept their finger on the texture throughout the block. The researcher offered participants a break at the end of each block whilst changing the stage’s orientation.

### 2.3. Recordings

EEG data were recorded continuously using a 129-channel sponge-based geodesic sensor net (Magstim EGI, UK). The net positioning was aligned to three anatomical landmarks, two preauricular points and the nasion. Electrode impedances were kept below 50 kΩ. A recording band-pass filter was set at 0.001–200 Hz with a sampling rate of 1,000 Hz. Electrode Cz was used as a reference electrode. The six forces and torques acting on the tile due to the finger touch were recorded using a Hopkinson Research six-axis sensor (Hopkinson, 2020), with a sampling rate of 1,000 Hz. Finger position in the XY plane was calculated from the block averaged (100 Hz) forces and torques.

### 2.4. Pre-processing

EEG pre-processing was conducted using BESA v 6.1 (MEGIS GmbH, Germany). Eye blinks and electrocardiographic artefacts were removed using principal component analysis (Berg and Scherg, 1994), which allowed the researcher to assess the feasibility of components for each participant and each experimental block. Data were filtered using 0.5 Hz high-pass and 100 Hz low-pass filters, with a notch filter (50 Hz ± 2 Hz). Data were visually inspected for the presence of any movement or muscle artefacts. Trials containing artefacts were excluded from subsequent analyses. EEG signals were downsampled to 256 Hz and re-referenced using the common average method (Lehmann, 1984).

Six-axis sensor data were cleaned and visually inspected using in-house software developed in Python 3 (Van Rossum and Drake, 2009). Data were epoched relative to the visual trial onset marker. Trials were rejected when 25% of samples were missed due to recording issues. Texture transition was calculated from position data from when the finger position crossed ±5 mm on the x-axis corresponding to the centre point of the sample which corresponds to a timepoint within the transition period from one texture to the other. Relative time from the visual trigger to the texture transition was calculated.

After EEG and six-axis sensor pre-processing were complete, the average number of trials for each condition across all participants was: smooth to rough, 39.57 ± 9.77; rough to smooth, 41.70 ± 7.78. The average number of accepted trials did not differ across conditions (p>0.05).

### 2.5. Analysis

Texture transition markers were computed relative to trial onset marker times, which were synchronised to EEG data. Data were epoched −2–2 s relative to the transition marker. The power spectra were computed in MATLAB (The MathWorks, Inc., United States) using Welch’s power spectral estimate method. The power spectral densities were computed from 1 s windows shifted in overlapping 0.01 s increments to yield a power time series of 400 points. Data were smoothed using a Hanning window. The power spectral densities were estimated in the range of 1–80 Hz with a frequency resolution of 1 Hz. *Z*-values were computed after the power calculation at each time bin using the median and median absolute deviation (MAD) across trials and conditions (Klimesch et al., 1998; Pfurtscheller, 1999; Arnal et al., 2015). Comparing the period texture change to an active baseline of texture processing during active touch allows for the isolation of the neural correlates associated with the phenomena of texture change detection during active touch. Therefore, the obtained z-transformed power values were evaluated prior to texture change (−650 – −200 ms) and during texture change (0–450 ms). The pre-transition period was selected as an active baseline instead of a pre-stimulus baseline with rest, as ongoing tactile perception provides a more naturalistic paradigm to investigate the complexities of sensory experience during perceptual change paradigms; this approach has been previously demonstrated in EEG research investigating auditory and visual change detection within complex scenes and environments (O’Connell et al., 2012; Kelly and O’Connell, 2013; Boubenec et al., 2017).

Permutation analyses with 5,000 repetitions were implemented using *statcond.m* from the EEGLAB library (Delorme and Makeig, 2004; Maris and Oostenveld, 2007). The permutation analysis was conducted as an exploratory data analysis across all 128 electrodes with power averaged over the time period and frequency band of interest. This approach helps to reduce the likelihood of Type 1 errors whilst improving the statistical power of the analysis (Maris, 2004; Maris and Oostenveld, 2007). Identified electrodes showing significant differences between conditions ( p<0.05 ) were grouped into clusters based on spatial neighbours and changes in absolute power *z*-scores subjected to paired samples t-test to investigate the direction of the effect. The results were corrected for multiple comparisons using the Bonferroni correction. Artefactual data were removed from identified electrodes where the *z*-score exceeded 5 MAD, in line with previous recommendations on identification of statistical outliers (Thatcher et al., 2019).

To assess the variability in the unilateral finger exploration, mean load (g) was computed for each pre-transition and transition period used in the EEG analysis. These data were then averaged over each participant and compared using paired samples *t*-test.

## 3. Results

### 3.1. Load

Mean values of total load were 30.45 ± 19.54 g (M ± SD) for the pre-transition period, and 50.44 ± 5.05 g during the transition period. A paired samples *t*-test demonstrated no significant difference between load in the two time periods.

### 3.2. EEG

Absolute power, normalised with a *z*-score transformation, was evaluated during pre-transition texture processing (−650 – −200 ms) and during texture transition (0–450 ms). Permutation analyses with 5,000 repetitions ( p<0.05 ) identified one contralateral electrode and a cluster of two ipsilateral electrodes which demonstrated statistically significant effects of texture change, regardless of texture change direction, when compared to pre-transition texture processing in alpha-band (8–12 Hz). Both were located over bilateral sensorimotor areas. Permutation analysis found no statistically significant electrodes for beta- (16–24 Hz) or theta-band (4–7 Hz) when comparing pre-transition texture processing to texture transition. Further, texture transition was then split by condition for comparison of oscillatory changes associated with smooth-to-rough transitions and *vice-versa*. When comparing the two conditions using permutation analysis, one central electrode demonstrated a statistically significant effect of direction of texture change during the transition period for beta-band, whereas no statistically significant effect was identified for alpha- or theta-band.

**Figure 2:**
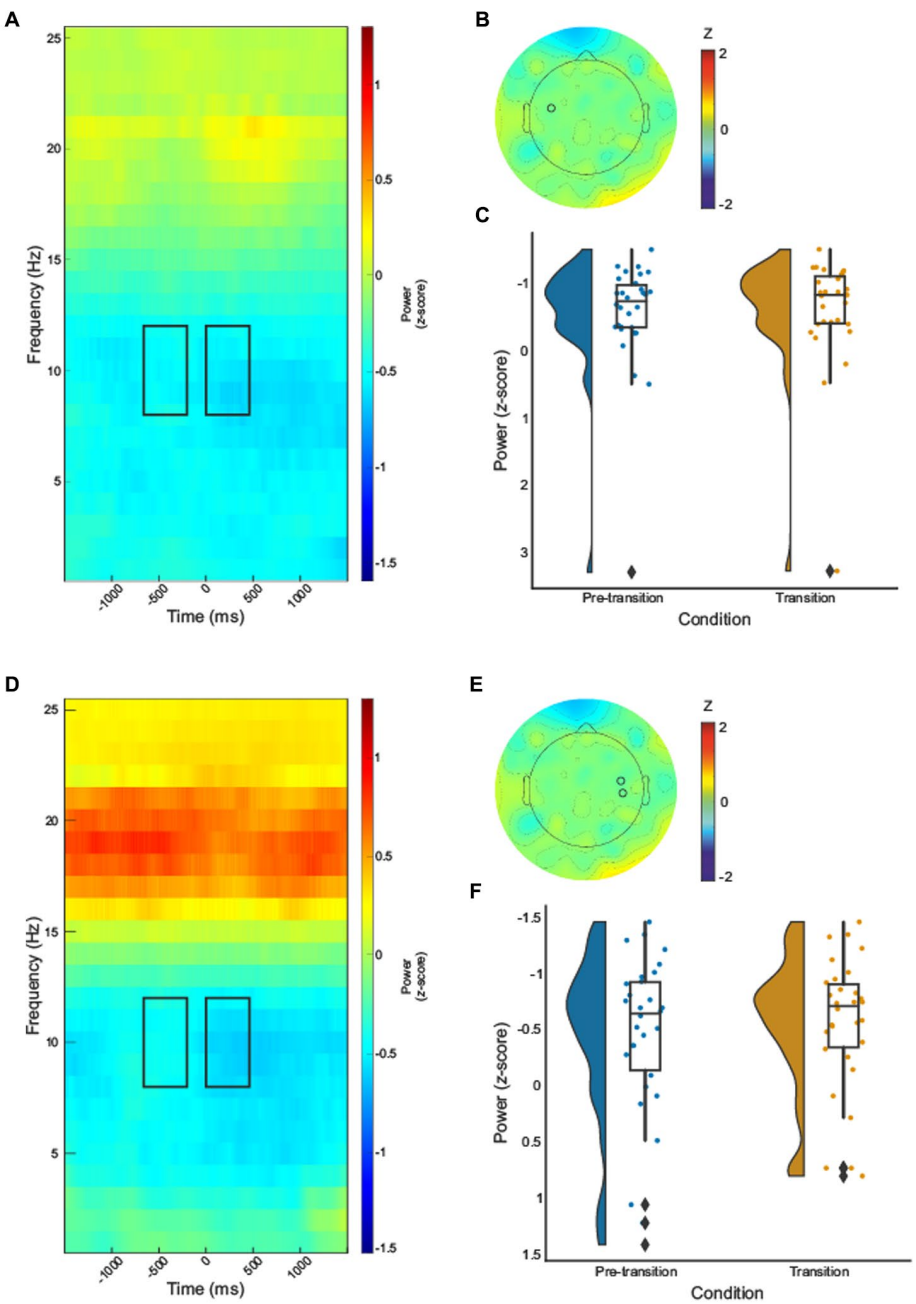
Time–frequency spectrograms for electrode 35 **(A)** and cluster one (electrodes 103 and 110) **(D)**, black boxes indicate the time (pre-transition texture processing −600 – −200, and transition processing 0–450 ms) and frequency (8–12Hz) where significant effects were identified. The half violin plots depict the probability distributions of the data in electrode 35 **(C)** and cluster one (electrodes 103 and 110) **(F)**. The individual dots show data points from each participant. The boxplots indicate the median, upper and lower quartiles, as well as the interquartile range (IQR) between the 25th and 75th percentile, whilst the whiskers represent scores outside of the IQR. Grand average topographic maps for the alpha-band are shown, with electrode 35 **(B)** and electrodes 110 and 103 **(E)** locations overlayed.

Paired samples *t*-tests were computed for electrodes identified by permutation analysis. A significant decrease in alpha-band power during texture change when compared to ongoing texture processing was revealed for the contralateral electrode identified (electrode 35), t(29)=3.57,p=0.001 , Figures 2A-C. A cluster of two electrodes over ipsilateral sensorimotor regions (110 and 103, C6 according to the 10–10 system; Luu and Ferree, 2005) were found to be significant, Figures 2D-F. Paired samples *t*-test demonstrated a significant decrease in alpha-band power during texture transition relative to pre-transition texture processing, t(29)=3.03,p=0.005 .

**Figure 3:**
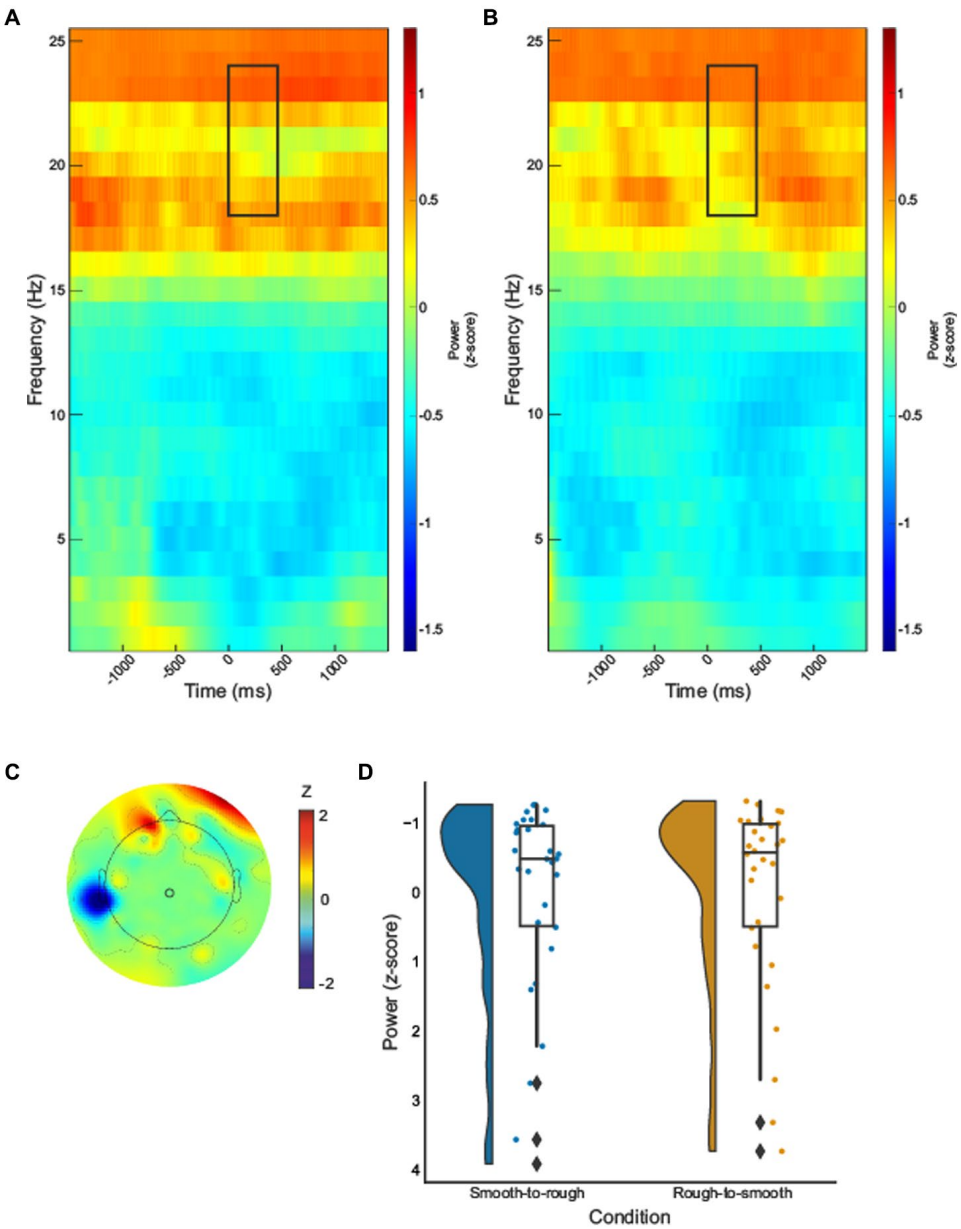
Time–frequency spectrograms for electrode 55 for smooth to rough transition **(A)** and rough to smooth transition **(B)**, black boxes indicate the time (0–450 ms) and frequency (16–24Hz) where statistically significant effects were identified. The half violin plots depict the probability distributions of the data in electrode 55 **(D)**. The individual dots show data points from each participant. The boxplots indicate the median, upper and lower quartiles, as well as the IQR between the 25th and 75th percentile, whilst the whiskers represent scores outside of the IQR. Grand average topographic maps for beta-band are shown, with electrode 55 location overlayed **(C)**.

In the beta-band, a statistically significant effect was identified over central sensorimotor regions in electrode 55, Figure 3. Transitioning from rough to smooth textures demonstrated a significant decrease in power when compared with transitioning from smooth to rough, t(29)=3.03,p=0.005 .

## 4. Discussion

The present study aimed to establish how the brain encodes texture change by assessing oscillatory differences in alpha-, beta- and theta-bands as the finger transitions from one texture to another during active touch exploration. Texture transition produced significant differences in alpha and beta frequency bands, whereas no statistically significant effects were observed in theta-band. In line with our hypothesis, alpha-band power over sensorimotor cortical regions decreased during texture transition when compared to pre-transition texture processing. Further, beta-band power decreased during texture transition when transitioning from rough-to-smooth textures over central regions, relative to smooth-to-rough trials, supporting previous findings from our lab (Henderson et al., 2022). Results indicate that perceptual texture change is observable in oscillatory brain activation patterns recorded by EEG under a naturalistic paradigm.

Bilateral decreases in alpha-band power were observed for texture transition relative to pre-transition texture processing over sensorimotor regions. Alpha-band power is associated with bilateral activation following tactile stimulation (Tamè et al., 2016; Genna et al., 2018), wherein a decrease in power, or attenuation of oscillations, signifies an increase in cortical processing (Cheyne et al., 2003; Gaetz and Cheyne, 2006). The present results indicate that it is likely that alpha-band oscillations also reflect perceptual texture change mechanisms in the brain, which manifests as increased cortical activation (decrease in power) to encode novel or changing tactile input from mechanoreceptors. Previous research with active touch has suggested that alpha-band power is also associated with roughness, wherein rougher textures increase cortical activation (Henderson et al., 2022), whilst passive touch research reports increased cortical activation for smoother textures (Ballesteros et al., 2009; Moungou et al., 2016; Genna et al., 2018). In contrast to our hypothesis, the present study demonstrated no significant effect in alpha-band when comparing rough to smooth transition and *vice-versa*. This suggests that the effects found in alpha-band for this study are representative of change detection, rather than roughness modulation. Therefore, it is possible that the role of alpha-and oscillations during change detection differs from that during roughness encoding in tactile stimulation, though further research is necessary to confirm this hypothesis.

Beta-band power was decreased over central sensorimotor regions when comparing transition from rough to smooth with the opposite condition. This supports our hypothesis that smooth textures increase cortical activation in the beta-band and accords with previous studies using passive (Moungou et al., 2016; Genna et al., 2018) as well as active touch paradigms (Henderson et al., 2022). The duplex theory states that the perception of fine textures is mediated by high-frequency vibrations from tactile elements (Katz, 1925, 1989; Hollins and Risner, 2000). Previous research suggests that beta-band activity is mediated by vibration intensity (Park et al., 2021), which contributes to observed difference in the EEG signal between rough and smooth textures (Henderson et al., 2022). Interestingly, the present study demonstrated that texture modulates beta-band power during transitioning between two textures, which, to the best of our knowledge, is the first instance demonstrating this effect.

Increased frontal theta-band oscillations have previously been shown to coincide with the enhancement of the sMMN component (Zhang et al., 2019). We hypothesised that such components may be visible in theta-band changes in time-frequency analysis. However, in this naturalistic active touch paradigm, perceptual texture change did not modulate theta-band power *per se*. Therefore, identification of change detection ERP components may not be detectable with the current temporal resolution (100 Hz) computation of time markers. Time-locking may need to be more precise to directly compare well known change perception ERPs, such as the sMMN, in ongoing time-frequency changes. However, this is the first known study to investigate change perception in active touch in a naturalistic paradigm, offering a more ecologically valid approach than previous oddball paradigms with stationary participants. Therefore, it is also possible that ERP correlates of change detection seen in previous studies differ, or are not present, when performing ongoing explorations of texture.

The present study used continuous tactile stimulation to investigate perceptual texture change in the way that we typically encounter it in our environment, as change occurring on top of an ongoing sensory experience (Gallace et al., 2006, 2007). Contrasting with rest improves the signal-to-noise ratio of post-stimulus activity (Toro et al., 1994). There was no rest period included in this paradigm, which incidentally reduces the signal-to-noise ratio and results in small effect sizes due to the comparison of brain oscillations with active baseline conditions. However, to understand the features of change detection, it was essential to compare texture change to ongoing tactile perception. Previous EEG research has investigated perceptual change detection using complex ongoing auditory (Boubenec et al., 2017) and visual stimuli (O’Connell et al., 2012; Kelly and O’Connell, 2013). Therefore, ongoing stimulation is appropriate for perceptual change paradigms, as they allow researchers to understand the complexities of sensory experience during more naturalistic tasks. Though future paradigms may also benefit from a period of pre-stimulus rest to improve the signal-to-noise ratio for additional analyses in tandem with an active baseline approach.

There are limitations to the present design. As a lab-based study, and due to the trial requirements for time-frequency EEG analysis (Cohen, 2016), participants were exposed to the two textured stimuli repeatedly over the course of the experiment. Therefore, it is likely that changes in texture were predictable, which may have developed expectations and diminished stimulus novelty. Further, repeated stimulation may have led to sensory desensitisation (Klingner et al., 2011; Graczyk et al., 2018) and reduced task engagement (Lelis-Torres et al., 2017). However, repeated trials are necessitated by the time-frequency method (Cohen, 2016). Future research could consider the use of one repetitive stimulus embedded within a more complex paradigm with various novel stimuli and various gradations of intensity to manipulate the degree to which the textural change was detectable whilst maintaining novelty, engagement and reducing desensitisation.

In conclusion, the present study demonstrates that alpha-band activity is related to perceptual texture change during continuous texture exploration, whilst beta-band may be linked to the processing of vibrotactile cues. Therefore, this study hypotheses that alpha- and beta-band both play a functional role in acting as a change detection mechanism as well as processing surface properties of the texture, respectively (Henderson et al., 2022). However, the neural underpinnings of texture processing require further elucidation. Future research should consider using active exploration of one repetitive stimulus alongside various novel stimuli, which will enable researchers to maintain novelty and test the hypothesis that alpha-band activity encodes perceptual texture change. To our knowledge, the results demonstrate for the first time that the encoding of textural change detection can be measured in the brain during ongoing active exploration of surfaces.

## Data availability statement

The datasets presented in this study can be found in online repositories. The names of the repository/repositories and accession number(s) can be found at: https://osf.io/s8xt6/?view_only=3c85a99cb2f948caa3439f2a7823b281.

## Ethics statement

The studies involving human participants were reviewed and approved by the study was approved by the Research Ethics Committee of the University of Liverpool. The patients/participants provided their written informed consent to participate in this study.

## Author contributions

JH: conceptualization, methodology, software, formal analysis, investigation, data curation, writing–original draft, writing–review and editing, visualisation, and project administration. TM, AN-F, and DH: investigation and writing–review and editing. AH: methodology, software, and writing–review and editing. TG: conceptualization, funding acquisition, and supervision. AM and AS: conceptualization, supervision, and writing–review and editing. NF: conceptualization, methodology, software, formal analysis, writing–review and editing, supervision, and funding acquisition. All authors contributed to the article and approved the submitted version.

## Funding

This work was supported by the EPSRC [Grant number: EP/S513830/1], and Unilever.

## Conflict of interest

The authors declare that the research was conducted in the absence of any commercial or financial relationships that could be construed as a potential conflict of interest.

## Publisher’s note

All claims expressed in this article are solely those of the authors and do not necessarily represent those of their affiliated organizations, or those of the publisher, the editors and the reviewers. Any product that may be evaluated in this article, or claim that may be made by its manufacturer, is not guaranteed or endorsed by the publisher.
